# Trends in hospitalizations for non-traumatic subarachnoid hemorrhage in the United States: 2012–2020

**DOI:** 10.1186/s12883-026-05200-0

**Published:** 2026-07-24

**Authors:** Joseph R. Geraghty, Connor A. Law, Rogan G. Magee, Ali G. Hamedani, Michelle Caunca, Andrea L.C. Schneider, Holly Elser

**Affiliations:** 1 Department of Neurology, University of Pennsylvania Perelman School of Medicine, Philadelphia, United States; 2 Department of Biostatistics, Epidemiology, and Informatics, University of Pennsylvania Perelman School of Medicine, Philadelphia, Pennsylvania United States; 3 Department of Neurology, University of California San Francisco, San Francisco, California United States

**Keywords:** Subarachnoid hemorrhage, National inpatient sample, Epidemiology

## Abstract

**Background:**

Although non-traumatic subarachnoid hemorrhage (SAH) remains an important cause of death and disability worldwide, contemporary research on hospitalization trends over time and across demographic groups remains scarce.

**Methods:**

We performed a cross-sectional study of U.S. SAH hospitalizations from 2012 to 2020 using data from the National Inpatient Sample. SAH-related hospital discharges were identified using diagnostic codes from the *International Classification of Disease*,* Ninth* and *Tenth Revisions* (ICD-9/10). Incidence per 100,000 hospitalizations was calculated (2012–2020), pre- and post- the ICD-9 to ICD-10 transition (2012–2015; 2016–2020), and yearly among all hospitalizations and in subgroups defined by patient age, sex, race and ethnicity, and hospital region. Linear regression models were used to estimate change in incidence over time (separately for 2012–2015 and 2016–2020). Estimates are reported per 100,000 hospitalizations.

**Results:**

From 2012 to 2020, the overall incidence of SAH hospitalization was 88.9 per 100,000 hospital discharges (95%CI = 88.6,89.3). We observed a discontinuity in incidence prior to (72.9, 95%CI = 72.5,73.5) and after (102.1, 95%CI = 101.6,102.5) the transition to ICD-10. From 2012 to 2015 the annual incidence of non-traumatic SAH hospitalization was relatively stable (3.6, 95%CI=-0.1,7.3), but higher among males, individuals of White, Black, and Asian or Pacific Islander race, and in the Midwest. From 2016 to 2020, the annual incidence of non-traumatic SAH hospitalization decreased (-2.9, 95%CI=-3.4,-2.3). This finding was largely consistent across age, sex, race or ethnicity, and region subgroups, with the largest decrease in incidence observed for aged ≥ 75 years (-8.4, 95%CI=-12.7,-4.2) and for individuals of Asian or Pacific Islander race (-11.3, 95%CI=-20.2,-2.4).

**Conclusions:**

Although the incidence of non-traumatic SAH hospitalization remained stable from 2012 to 2015 and decreased from 2016 to 2020, our analysis suggests persistent differences in the risk of SAH hospitalization across subgroups defined by age, sex, and race/ethnicity.

**Supplementary Information:**

The online version contains supplementary material available at https://doi.org/10.1186/s12883-026-05200-0.

## Introduction

Non-traumatic subarachnoid hemorrhage (SAH), most commonly from rupture of cerebral aneurysms, is a neurological and neurosurgical emergency with high morbidity and mortality, accounting for 5–10% of all strokes [1, 2]. Studies using global data have suggested that while age-standardized rates of SAH have decreased over the last few decades, SAH remains a an important cause of premature morbidity and mortality [3].

Contemporary data that examines overall trends in non-traumatic SAH hospitalizations in the United States (U.S.), as well as trends within key patient subgroups, remain limited and are needed to clarify how the burden of hospitalization for non-traumatic SAH is distributed over time, and across demographic and regional patient subgroups. The present study therefore leverages nationally representative data for U.S. hospitalizations from the National Inpatient Survey (NIS) from 2012 to 2020. Our analysis aims to characterize trends in hospitalizations for non-traumatic SAH overall, and within key demographic subgroups defined by age, sex, socially constructed race and ethnicity, and U.S. Census Region. Understanding temporal trends in SAH hospitalization can provide context for national surveillance efforts and identify demographic groups that continue to experience disproportionate disease burden.

## Methods

### Study design and population

We conducted a cross-sectional study of the incidence of non-traumatic SAH hospitalizations in the U.S. from 2012 to 2020. We used data from the NIS, the largest publicly available, all-payer inpatient healthcare database in the U.S [4]. The NIS data are made available through the Healthcare Cost and Utilization Project (HCUP), sponsored by the Agency for Healthcare Research and Quality. From 2012 onward, the NIS is a 20% sample of all U.S. hospitalizations, including unweighted data from approximately 7 million hospitalizations per year. Available admission-level data include patient demographics, diagnoses, inpatient procedures, and hospital characteristics. This study is reported in accordance with the Strengthening of Reporting in Observational Epidemiology (STROBE) guidelines [5].

### Non-traumatic subarachnoid hemorrhage hospital discharges

We identified all hospital discharges for non-traumatic SAH using diagnostic codes from the International Classification of Diseases, Ninth Revision (ICD-9) prior to October 2015 and the Tenth Revision (ICD-10) thereafter (Table S1) (hereafter “hospitalizations”). While ICD-9 included a single code for non-traumatic SAH, ICD-10 expanded to multiple codes for SAH based on blood vessel involved and symptoms. Our analysis was restricted to those hospitalizations in which a code for SAH was in the first diagnostic position (i.e., principal hospital diagnosis). We did not include hospitalizations where the primary diagnosis involved intracranial trauma or other forms of brain hemorrhage. In sensitivity analysis, we excluded persons with a secondary diagnosis of traumatic brain injury (TBI) (Table S2).

### Covariates

Patient- and hospital-level characteristics included categorical age (< 24 years; 25–49 years; 50–74 years; ≥75 years; missing), sex (male; female; missing), socially constructed race and ethnicity (White; Black; Hispanic; Asian or Pacific Islander; Native American; Other; missing), hospital region (Northeast; South; Midwest and North Central; West), and calendar year. For analyses, race and ethnicity were defined using the HCUP unform variable, in which categories are considered mutually exclusive and ethnicity takes precedence over race in setting the HCUP uniform value when both are reported.

### Statistical analysis

All statistical analyses incorporated sampling weights to account for the complex survey design of NIS, which allows estimates reported herein to be nationally representative of all U.S. hospitalizations. The annual incidence per 100,000 hospital discharges (95% confidence intervals [CIs]) were calculated assuming a Poisson distribution for 2012–2020. We first used linear regression models to estimate the change in the incidence of non-traumatic SAH hospitalization for 2012–2015 and 2016–2020 separately, as prior research has suggested that the transition from ICD-9 to ICD-10 in October 2015 influenced estimates of hospitalizations for several neurological and neurosurgical conditions [6], including non-traumatic SAH. As a secondary analysis, we repeated our main analysis of trends in non-traumatic SAH hospitalization within population subgroups defined by patient age, sex, race and ethnicity, and U.S. Census Region.

Our analysis further included the following robustness checks. First, we used joinpoint regression, a modeling approach that allows for estimation of time-specific trends from aggregated data including complex survey data, to characterize trends in non-traumatic SAH hospitalizations before and after the ICD transition [7]. Joinpoints for regression analysis are selected using a data-driven approach based on weighted Bayesian information criterion [8]. Second, we repeated our main analysis from 2012 to 2019, excluding the year 2020 as prior research establishes global declines in the overall volume of stroke hospitalizations, including admissions for intracranial hemorrhage, during the COVID-19 pandemic [9–11]. Third, we repeated our main analysis excluding the ICD-10 codes I69 (corresponding to sequelae following SAH) and I62.9 (nontraumatic intracranial hemorrhage, unspecified). Finally, because the entity of interest in this analysis is non-traumatic SAH, we repeated our main analysis excluding hospitalizations with a secondary diagnosis of TBI according to the case definition from the Centers for Disease Control and Prevention (CDC) [12–14].

The incidence of non-traumatic SAH hospitalizations is reported throughout per 100,000 hospital discharges. Statistical analyses were conducted using R Statistical Software version 4.3.2 (R Foundation for Statistical Computing, Vienna, Austria) and Joinpoint Trend Analysis Software version 5.0.3 (National Cancer Institute). Statistical significance was defined as a two-sided *p*-value < 0.05.

## Results

**Fig. 1:**
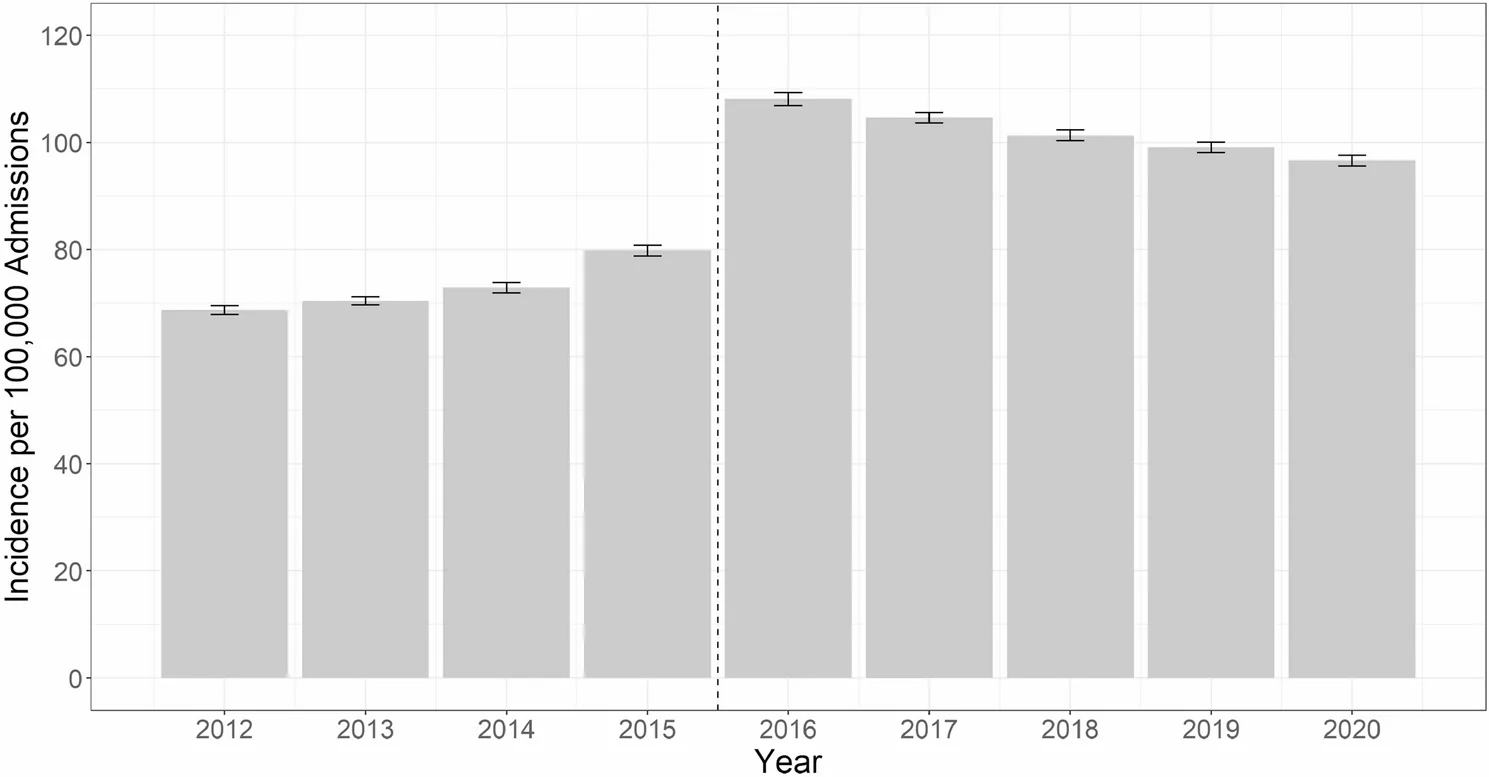
Annual incidence of non-traumatic subarachnoid hemorrhage hospitalizations per 100,000 discharges (standard error) in the National Inpatient Sample (NIS), 2012–2020. Given the ICD-9 to ICD-10 transition in October 2015, the dashed line demarcates before (2012–2015) and after (2016–2020) the transition

The incidence of non-traumatic SAH hospitalizations in the U.S. from 2012 to 2020 was 88.9 per 100,000 hospitalizations (95% CI: 88.6, 89.3) (Table 1). There was a large increase in the estimated incidence of non-traumatic SAH hospitalization after (102.1 in 2016, 95% CI: 101.6, 102.5) compared with prior to (72.9 in 2015, 95% CI: 72.5, 73.5) the transition to ICD-10 (Fig. 1). Evaluating trends over time, the incidence of non-traumatic SAH hospitalization remained stable from 2012 to 2015 (3.6, 95% CI: -0.1, 7.3) and decreased from 2016 to 2020 (-2.9, 95% CI: -3.4, -2.3) (Table S3).

**Table 1:** Incidence of non-traumatic subarachnoid hemorrhage hospitalizations per 100,000 discharges (95% confidence interval) in the National Inpatient Sample (NIS), 2012–2020*

	2012–2020	2012–2015	2016–2020
Overall	88.9 (88.6, 89.3)	72.9 (72.5, 73.4)	102.1 (101.6, 102.5)
Age			
≤24 years	10.0 (9.7, 10.2)	10.2 (9.8, 10.5)	9.8 (9.4, 10.1)
25–49 years	79.9 (79.2, 80.5)	72.5 (71.6, 73.4)	85.9 (85.0, 86.8)
50–74 years	135.2 (134.5, 135.9)	114.8 (113.9, 115.8)	151.2 (150.2, 152.1)
≥75 years	98.5 (97.7, 99.2)	68.4 (67.4, 69.3)	122.7 (121.6, 123.9)
Missing	55.5 (37.1, 73.8)	59.6 (38.3, 80.9)	39.2 (4.8, 73.6)
Sex			
Male	84.6 (84.1, 85.1)	67.6 (66.9, 68.2)	98.1 (97.4, 98.8)
Female	92.3 (91.9, 92.7)	76.9 (76.3, 77.5)	105.2 (104.6, 105.9)
Missing	41.6 (26.7, 56.5)	49.1 (24.2, 73.9)	36.1 (17.8, 54.3)
Race or Ethnicity			
White	83.9 (83.5, 84.3)	68.9 (68.4, 69.5)	96.2 (95.6, 96.8)
Black	92.4 (91.5, 93.3)	74.2 (73.1, 75.4)	106.6 (105.3, 107.8)
Hispanic	90.9 (89.9, 91.8)	72.7 (71.4, 74.0)	104.9 (103.5, 106.3)
Asian or Pacific Islander	140.0 (137.6, 142.5)	115.8 (112.4, 119.2)	157.9 (154.5, 161.3)
Native American	79.5 (75.6, 83.3)	65.2 (59.8, 70.5)	90.5 (85.0, 96.0)
Other	108.9 (106.9, 110.8)	94.9 (92.2, 97.7)	120.0 (117.2, 122.8)
Missing	95.4 (93.8, 96.9)	81.1 (79.2, 83.0)	113.3 (110.8, 115.8)
Region			
Northeast	83.2 (82.5, 83.9)	68.9 (67.9, 69.9)	95.3 (94.2, 96.4)
Midwest	80.8 (80.2, 81.5)	69.4 (68.5, 70.3)	90.3 (89.4, 91.3)
South	89.8 (89.3, 90.3)	72.4 (71.7, 73.1)	103.8 (103.1, 104.6)
West	101.7 (100.9, 102.5)	81.8 (80.7, 82.8)	117.8 (116.7, 118.9)

*Given the ICD-9 to ICD-10 transition in October 2015, data is also presented before (2012–2015) and after (2016–2020) the transition

**Fig. 2:**
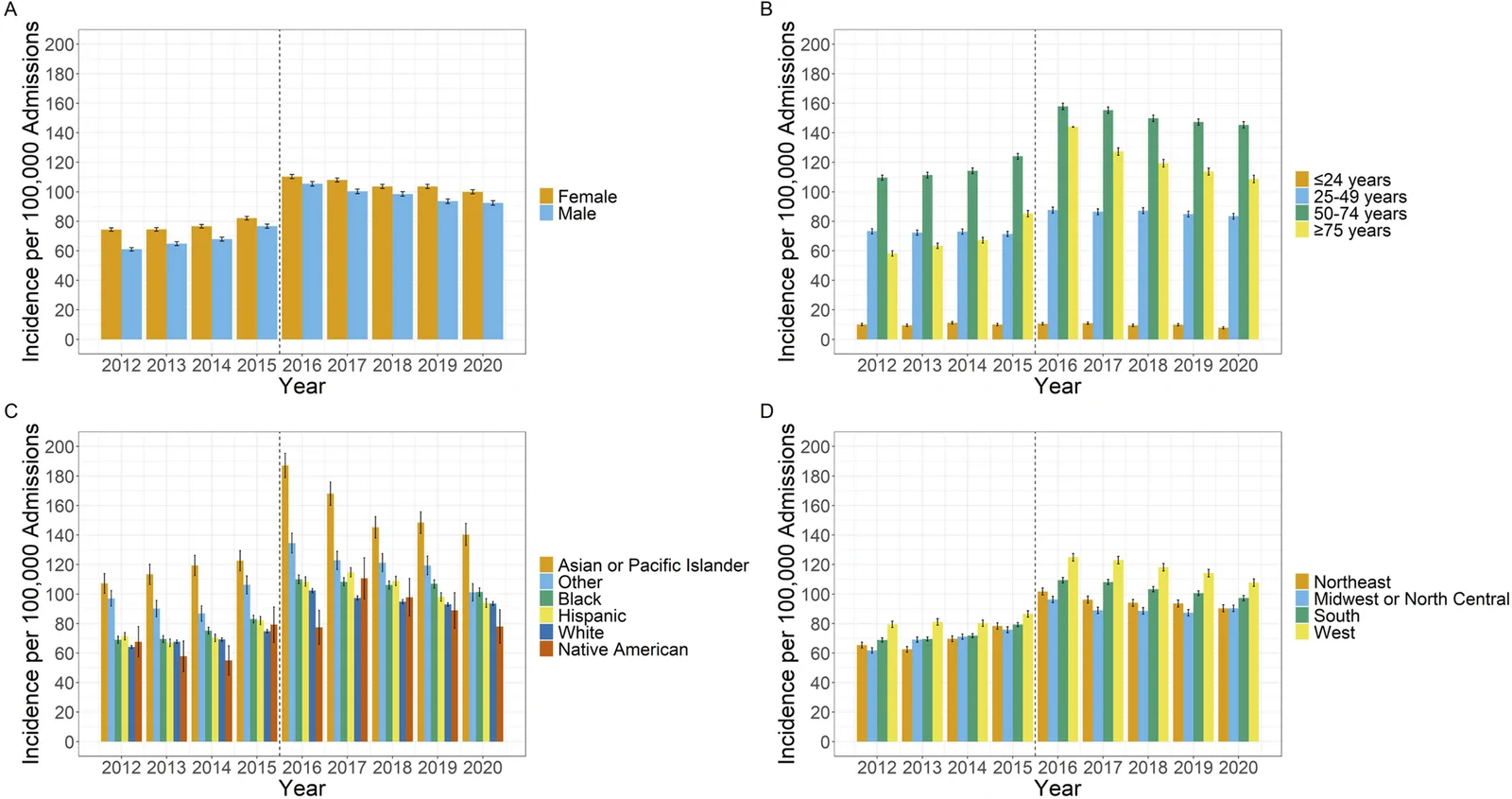
Annual incidence of non-traumatic subarachnoid hemorrhage hospitalizations per 100,000 discharges (standard error) in the National Inpatient Sample (NIS), 2012-2020*, stratified by sex (**A**), age (**B**), race or ethnicity (**C**), and region of the country (**D**). *Given the ICD-9 to ICD-10 transition in October 2015, the dashed line demarcates before (2012-2015) and after (2016-2020) the transition

The incidence of non-traumatic SAH hospitalization was consistently higher among individuals aged ≥ 25 years compared to < 25 years, with the highest incidence among individuals aged 50–74 years. Hospitalizations were more frequent among individuals of Asian or Pacific Islander race, Black race, and Hispanic ethnicity compared to White race, and in the West compared to the Northeast (Fig. 2). We did not observe clear differences in non-traumatic SAH hospitalization by sex. The annual change in non-traumatic SAH hospitalizations among demographic and hospital subgroups was largely stable from 2012 to 2015, but increased among males (5.0, 95% CI: 1.1, 8.9), individuals of White (3.4, 95% CI: 1.0, 5.7), Black (4.8, 95% CI: 0.3, 9.8), and Asian or Pacific Islander (5.2, 95% CI: 3.1, 7.4) race, and in the Midwest (4.4, 95% CI: 1.4, 7.4) (Table S4). From 2016 to 2020, annual rates of non-traumatic SAH hospitalizations decreased in most patient and hospital subgroups, with the largest annual change observed for aged ≥ 75 years (8.4, 95% CI: -12.7, -4.2) and for individuals of Asian or Pacific Islander race (-11.3, 95% CI: -20.2, -2.4).

In sensitivity analysis with data-driven Joinpoint selection, we observed an increase in the incidence of non-traumatic SAH hospitalization from 2012 to 2017 (10.3, 95%CI: 2.4,18.7; *p* = 0.02) with little change from 2017 to 2020 (-3.2, 95%CI: -16.3,11.9), with the increase in incidence estimated for the first epoch likely reflecting the increase in incidence observed after the ICD transition in October of 2015. Findings from joinpoint regression analysis were generally consistent across patient subgroups defined by sex, age, race and ethnicity, and U.S. Census Region (Table S5). The overall estimated incidence of SAH hospitalizations was robust to restriction of the study period to years 2012–2019 (88.1, 95%CI: 87.7,88.4), to the exclusion of ICD-10 codes I69 and I62.9 (89.0, 95%CI: 88.2,89.7), and to the exclusion persons with a diagnosis of TBI (89.0, 95%CI: 88.2,89.7) with consistent finding across the epochs of 2012–2015 and 2016–2020 (Tables S6–S8).

## Discussion

The incidence of non-traumatic SAH hospitalizations was stable from 2012 to 2015 and decreased from 2016 to 2020. Our results further suggest the incidence of non-traumatic SAH hospitalization was consistently elevated among persons aged 50–74 years and persons identified as Asian or Pacific Islander, Black, or Hispanic. Collectively, these findings underscore that key population subgroups experience persistently elevated incidence of non-traumatic SAH hospitalization that persisted despite changes in administrative coding. These findings may reflect differences in the distribution of established, modifiable risk factors for SAH, particularly hypertension and tobacco use [15, 16], within these populations.

Our study expands upon prior work in several ways. First, large-scale studies often treat SAH and intracerebral hemorrhage as a compositive outcome, which precludes understanding of trends for SAH alone [17, 18]. Further, much of the prior literature does not explicitly account for changes in coding practices surrounding the ICD transition when reporting changes in SAH hospitalizations or mortality rates [3, 19–22]. Additionally, many contemporary studies include age, sex, and race and ethnicity as baseline demographic covariates, but do not analyze temporal trends across these key patient subgroups. For example, whereas Wahood and colleagues evaluated aneurysmal SAH admissions and outcomes through 2018, our study examined all non-traumatic SAH hospitalizations through 2020, explicitly evaluated coding-era discontinuity, and characterized temporal trends across demographic and regional subgroups [23]. Our data further highlights both racial and geographical disparities in SAH care across the United States [24, 25]. These distinctions help to strengthen interpretation and add to the evidentiary base for secular trends in SAH hospitalizations in the U.S.

Notably, our analysis indicates a substantial increase in the incidence of SAH hospitalizations between 2015 and 2016, which corresponds to the transition from ICD-9 to ICD-10 in October 2015. Prior studies have looked at the impact of the ICD code transition period on coding practices for hospitalization diagnoses [6, 26, 27]. In the change from ICD-9 to ICD-10, the total number of diagnostic codes increased from approximately 15,000 to over 60,000, with several conditions having a large expansion in the number of diagnosis codes, including SAH [28]. Several studies have shown increased prevalence of non-neurological and neurological conditions that can be attributed to changes in coding practices [6, 26, 27]. One study of SAH hospitalizations between 2014 and 2015 and 2016–2017 showed a small but significant increase of 20 hospitalizations per month after the ICD code transition [6]. Our data expands upon this by analyzing a larger period before and after the coding transition. We show that while the transition period itself was associated with a significant absolute increase in non-traumatic SAH hospitalizations, this was followed by a decrease between 2016 and 2020.

While administrative data cannot establish whether declines in SAH hospitalizations following the ICD-10 transition represent underlying trends in SAH incidence at the population level, although several factors may have contributed to declines in SAH hospitalizations. First, this pattern may reflect continued secular declines in SAH–supported by reports in other population-based and global burden studies [3, 23]–potentially reflecting improved management of vascular risk factors, including hypertension and tobacco use [29] Second, changes in aneurysm detection, surveillance, and prophylactic treatment may have altered population risk for aneurysmal rupture [30] Third, as stated above, evolving coding practices after implementation of ICD-10 may have contributed to the observed decline, particularly if early ICD-10 adoption temporarily increased SAH coding capture. Our findings also demonstrate that analyses spanning the ICD-9 to ICD-10 transition require careful interpretation, providing methodological guidance for future studies using administrative databases–although importantly we note that differences across patient demographic subgroups persisted over nearly a decade despite coding-era discontinuities. Thus, the post-2016 decline should be interpreted cautiously as a hospitalization trend that is consistent with, but not definitive evidence of, declining SAH incidence.

A key contribution of this study is examination of trends in SAH hospitalization across patient subgroups defined by age, sex, race and ethnicity, and U.S. Census Region. By age, the incidence of SAH hospitalizations was highest among those ages 50–74 years, a finding that is in keeping with prior studies that demonstrate SAH incidence peaks in middle age [31, 32]. In subgroup analysis by sex, we observed similar rates of SAH hospitalization among men and women. As prior literature regarding differences in SAH risk by sex and gender remain mixed [33–36], future research that explores potential differences while incorporating information on the distribution of established vascular risk factors for SAH by sex and gender may be helpful. The present study further identifies regional differences in SAH hospitalization risk, with persistently elevated incidence observed in the Western U.S. compared with the Northeast, Southeast, and Midwest. While some prior work highlights differences in clinical care and outcomes of SAH patients in the U.S. [37], research that characterizes regional differences in SAH hospitalizations remains limited [38–40]. Future work that explores area-level differences in SAH incidence and that specifically considers the relative contributions of demographic differences, differences in vascular risk factors, and differences in billing practices is needed.

Finally, there are limited prior studies on racial/ethnic disparities in acute hemorrhagic stroke [18]. Hospitalizations for SAH in our study were greatest among patients identifying as Asian or Pacific Islander, which may be consistent with prior literature showing that Asian-Americans have higher rates of uncontrolled hypertension compared to non-Hispanic White people [41]. Further, smoking rates have remained stable in Asian-Americans while declining over time in other groups; however, this data may be under-reported [42]. This is also consistent with reports of higher SAH mortality rates in non-Hispanic Asian or Pacific Islanders based on data from the CDC WONDER database [19]. Similarly, the greater incidence in SAH hospitalizations among Black and Hispanic patients may also reflect greater rates of uncontrolled hypertension in these cohorts and lower odds of treatment of unruptured intracranial aneurysms [20, 41]. Importantly, Native American patients remain substantially underrepresented in the stroke literature, especially SAH [43]. Although the incidence of SAH hospitalizations observed in this study was lower than in several other minoritized subgroups, these nationally representative estimates provide useful descriptive data for an understudied population. Future work should aim to examine how contextual-level risk factors (e.g., neighborhood characteristics, environmental exposures, public health policies) and individual-level social factors (e.g., socioeconomic status, social networks) drive these differences.

### Limitations

We note several limitations related to the present study. First, the transition from ICD-9 to ICD-10 required us to perform separate analyses from 2012 to 2015 and from 2016 to 2020 due to the large absolute increase in non-traumatic SAH diagnoses that occurred between 2015 and 2016. We aimed to rigorously model discrete trends across these two epochs with Joinpoint regression, although data-driven joinpoint were generally identified in 2016 or 2017 after the ICD transition in 2015, making direct comparison with our primary analysis difficult.

Second, the NIS does not permit identification of individual patients; therefore, we could not distinguish incident SAH hospitalizations from readmissions. Prior longitudinal studies suggest that hospital readmission after stroke, including SAH, is common, particularly within the first several months after index hospitalization, although many readmissions are attributable to cardiovascular or other medical complications rather than recurrent hemorrhage [17, 44]. Consequently, our findings should be interpreted as trends in SAH-related hospitalizations rather than incident SAH events. Third, because estimates were calculated using a sample of hospital discharges as the denominator, SAH hospitalization incidence may be influenced by sampling or trends in hospitalizations more broadly.

The study period for this analysis extends through 2020, and thus interpretation of our findings is potentially complicated by previously-documented declines in stroke hospitalizations during the COVID-19 pandemic [45]. Reassuringly our results were robust to re-analysis excluding the year 2020 with follow-up extending from 2012 to 2019. Further, our analysis does not include more recent years of follow-up (e.g., beyond 2020). While the trends in non-traumatic SAH hospitalization may not be extrapolated beyond the study period, we note that differences observed across demographic subgroups are persistent throughout the study period. Because our analysis relies on diagnostic codes to identify non-traumatic SAH hospitalizations, we were unable to examine differences in the severity of non-traumatic SAH in hospitalized persons over time or across subgroups [23, 46].

Finally, the focus of this analysis is on trends in non-traumatic SAH hospitalizations over time and across population subgroups, rather than on risk factors for SAH hospitalizations. Further, several key vascular risk factors–including dietary patterns, body mass index, levels of physical activity, and alcohol and tobacco use–are often incompletely captured with diagnostic codes. Future research that incorporates information on established vascular risk factors for non-traumatic SAH may help to elucidate apparent, persistent differences in non-traumatic SAH hospitalizations across subgroups.

## Conclusion

Using a representative sample of nationwide hospitalization in the U.S. from 2012 to 2020, we find the incidence of non-traumatic SAH hospitalization remained stable from 2012 to 2015 and decreased from 2016 to 2020. Our analysis further suggests that key population subgroups experience persistently elevated risk for SAH hospitalization despite changes in administrative coding. These findings provide important context for future epidemiologic studies using administrative databases and identify demographic populations that may benefit from targeted prevention strategies.

## Supplementary Information

Supplementary Material 1.

## Data Availability

National Inpatient Sample (NIS) data are publicly available through the Agency for Healthcare Research and Quality Healthcare Cost & Utilization Project.
