# Supplementary material for: Trends in hospitalizations for non-traumatic subarachnoid hemorrhage in the United States: 2012–2020
Source: BMC Neurol. 2026 Jul 24;26:658. doi: 10.1186/s12883-026-05200-0 (PMC13681771; doi:10.1186/s12883-026-05200-0)
Supplement: Supplementary file 1 [file 12883_2026_5200_MOESM1_ESM.docx]

**Table S1.** ICD-9 and ICD-10 codes for non-traumatic subarachnoid hemorrhage.

| **Revision** | **Code** | **Description** |
| --- | --- | --- |
| ICD-9 | 430 | Subarachnoid hemorrhage |
| ICD-10 | I6000 | Nontraumatic subarachnoid hemorrhage from unspecified carotid siphon and bifurcation |
| ICD-10 | I6001 | Nontraumatic subarachnoid hemorrhage from right carotid siphon and bifurcation |
| ICD-10 | I6002 | Nontraumatic subarachnoid hemorrhage from left carotid siphon and bifurcation |
| ICD-10 | I6010 | Nontraumatic subarachnoid hemorrhage from unspecified middle cerebral artery |
| ICD-10 | I6011 | Nontraumatic subarachnoid hemorrhage from right middle cerebral artery |
| ICD-10 | I6012 | Nontraumatic subarachnoid hemorrhage from left middle cerebral artery |
| ICD-10 | I602 | Nontraumatic subarachnoid hemorrhage from anterior communicating artery |
| ICD-10 | I6020 | Nontraumatic subarachnoid hemorrhage from unspecified anterior communicating artery |
| ICD-10 | I6021 | Nontraumatic subarachnoid hemorrhage from right anterior communicating artery |
| ICD-10 | I6022 | Nontraumatic subarachnoid hemorrhage from left anterior communicating artery |
| ICD-10 | I6030 | Nontraumatic subarachnoid hemorrhage from unspecified posterior communicating artery |
| ICD-10 | I6031 | Nontraumatic subarachnoid hemorrhage from right posterior communicating artery |
| ICD-10 | I6032 | Nontraumatic subarachnoid hemorrhage from left posterior communicating artery |
| ICD-10 | I604 | Nontraumatic subarachnoid hemorrhage from basilar artery |
| ICD-10 | I6050 | Nontraumatic subarachnoid hemorrhage from unspecified vertebral artery |
| ICD-10 | I6051 | Nontraumatic subarachnoid hemorrhage from right vertebral artery |
| ICD-10 | I6052 | Nontraumatic subarachnoid hemorrhage from left vertebral artery |
| ICD-10 | I606 | Nontraumatic subarachnoid hemorrhage from other intracranial arteries |
| ICD-10 | I607 | Nontraumatic subarachnoid hemorrhage from unspecified intracranial artery |
| ICD-10 | I608 | Other nontraumatic subarachnoid hemorrhage |
| ICD-10 | I609 | Nontraumatic subarachnoid hemorrhage, unspecified |
| ICD-10 | I629 | Nontraumatic intracranial hemorrhage, unspecified |
| ICD-10 | I6900 | Unspecified sequelae of nontraumatic subarachnoid hemorrhage |
| ICD-10 | I6901 | Cognitive deficits following nontraumatic subarachnoid hemorrhage |
| ICD-10 | I69010 | Attention and concentration deficit following nontraumatic subarachnoid hemorrhage |
| ICD-10 | I69011 | Memory deficit following nontraumatic subarachnoid hemorrhage |
| ICD-10 | I69012 | Visuospatial deficit and spatial neglect following nontraumatic subarachnoid hemorrhage |
| ICD-10 | I69013 | Psychomotor deficit following nontraumatic subarachnoid hemorrhage |
| ICD-10 | I69014 | Frontal lobe and executive function deficit following nontraumatic subarachnoid hemorrhage |
| ICD-10 | I69015 | Cognitive social or emotional deficit following nontraumatic subarachnoid hemorrhage |
| ICD-10 | I69018 | Other symptoms and signs involving cognitive functions following nontraumatic subarachnoid hemorrhage |
| ICD-10 | I69019 | Unspecified symptoms and signs involving cognitive functions following nontraumatic subarachnoid hemorrhage |
| ICD-10 | I69020 | Aphasia following nontraumatic subarachnoid hemorrhage |
| ICD-10 | I69021 | Dysphasia following nontraumatic subarachnoid hemorrhage |
| ICD-10 | I69022 | Dysarthria following nontraumatic subarachnoid hemorrhage |
| ICD-10 | I69023 | Fluency disorder following nontraumatic subarachnoid hemorrhage |
| ICD-10 | I69028 | Other speech and language deficits following nontraumatic subarachnoid hemorrhage |
| ICD-10 | I69031 | Monoplegia of upper limb following nontraumatic subarachnoid hemorrhage affecting right dominant side |
| ICD-10 | I69032 | Monoplegia of upper limb following nontraumatic subarachnoid hemorrhage affecting left dominant side |
| ICD-10 | I69033 | Monoplegia of upper limb following nontraumatic subarachnoid hemorrhage affecting right non-dominant side |
| ICD-10 | I69034 | Monoplegia of upper limb following nontraumatic subarachnoid hemorrhage affecting left non-dominant side |
| ICD-10 | I69039 | Monoplegia of upper limb following nontraumatic subarachnoid hemorrhage affecting unspecified side |
| ICD-10 | I69041 | Monoplegia of lower limb following nontraumatic subarachnoid hemorrhage affecting right dominant side |
| ICD-10 | I69042 | Monoplegia of lower limb following nontraumatic subarachnoid hemorrhage affecting left dominant side |
| ICD-10 | I69043 | Monoplegia of lower limb following nontraumatic subarachnoid hemorrhage affecting right non-dominant side |
| ICD-10 | I69044 | Monoplegia of lower limb following nontraumatic subarachnoid hemorrhage affecting left non-dominant side |
| ICD-10 | I69049 | Monoplegia of lower limb following nontraumatic subarachnoid hemorrhage affecting unspecified side |
| ICD-10 | I69051 | Hemiplegia and hemiparesis following nontraumatic subarachnoid hemorrhage affecting right dominant side |
| ICD-10 | I69052 | Hemiplegia and hemiparesis following nontraumatic subarachnoid hemorrhage affecting left dominant side |
| ICD-10 | I69053 | Hemiplegia and hemiparesis following nontraumatic subarachnoid hemorrhage affecting right non-dominant side |
| ICD-10 | I69054 | Hemiplegia and hemiparesis following nontraumatic subarachnoid hemorrhage affecting left non-dominant side |
| ICD-10 | I69059 | Hemiplegia and hemiparesis following nontraumatic subarachnoid hemorrhage affecting unspecified side |
| ICD-10 | I69061 | Other paralytic syndrome following nontraumatic subarachnoid hemorrhage affecting right dominant side |
| ICD-10 | I69062 | Other paralytic syndrome following nontraumatic subarachnoid hemorrhage affecting left dominant side |
| ICD-10 | I69063 | Other paralytic syndrome following nontraumatic subarachnoid hemorrhage affecting right non-dominant side |
| ICD-10 | I69064 | Other paralytic syndrome following nontraumatic subarachnoid hemorrhage affecting left non-dominant side |
| ICD-10 | I69065 | Other paralytic syndrome following nontraumatic subarachnoid hemorrhage, bilateral |
| ICD-10 | I69069 | Other paralytic syndrome following nontraumatic subarachnoid hemorrhage affecting unspecified side |
| ICD-10 | I69090 | Apraxia following nontraumatic subarachnoid hemorrhage |
| ICD-10 | I69091 | Dysphagia following nontraumatic subarachnoid hemorrhage |
| ICD-10 | I69092 | Facial weakness following nontraumatic subarachnoid hemorrhage |
| ICD-10 | I69093 | Ataxia following nontraumatic subarachnoid hemorrhage |
| ICD-10 | I69098 | Other sequelae following nontraumatic subarachnoid hemorrhage |
| ICD-10 | I6010 | Nontraumatic subarachnoid hemorrhage from unspecified middle cerebral artery |
| ICD-10 | I6011 | Nontraumatic subarachnoid hemorrhage from right middle cerebral artery |
| ICD-10 | I6012 | Nontraumatic subarachnoid hemorrhage from left middle cerebral artery |
| ICD-10 | I602 | Nontraumatic subarachnoid hemorrhage from anterior communicating artery |
| ICD-10 | I6020 | Nontraumatic subarachnoid hemorrhage from unspecified anterior communicating artery |
| ICD-10 | I6021 | Nontraumatic subarachnoid hemorrhage from right anterior communicating artery |
| ICD-10 | I6022 | Nontraumatic subarachnoid hemorrhage from left anterior communicating artery |
| ICD-10 | I6030 | Nontraumatic subarachnoid hemorrhage from unspecified posterior communicating artery |
| ICD-10 | I6031 | Nontraumatic subarachnoid hemorrhage from right posterior communicating artery |
| ICD-10 | I6032 | Nontraumatic subarachnoid hemorrhage from left posterior communicating artery |
| ICD-10 | I604 | Nontraumatic subarachnoid hemorrhage from basilar artery |
| ICD-10 | I6050 | Nontraumatic subarachnoid hemorrhage from unspecified vertebral artery |
| ICD-10 | I6051 | Nontraumatic subarachnoid hemorrhage from right vertebral artery |
| ICD-10 | I6052 | Nontraumatic subarachnoid hemorrhage from left vertebral artery |
| ICD-10 | I606 | Nontraumatic subarachnoid hemorrhage from other intracranial arteries |
| ICD-10 | I607 | Nontraumatic subarachnoid hemorrhage from unspecified intracranial artery |
| ICD-10 | I608 | Other nontraumatic subarachnoid hemorrhage |
| ICD-10 | I609 | Nontraumatic subarachnoid hemorrhage, unspecified |
| ICD-10 | I629 | Nontraumatic intracranial hemorrhage, unspecified |
| ICD-10 | I6900 | Unspecified sequelae of nontraumatic subarachnoid hemorrhage |
| ICD-10 | I6901 | Cognitive deficits following nontraumatic subarachnoid hemorrhage |
| ICD-10 | I69010 | Attention and concentration deficit following nontraumatic subarachnoid hemorrhage |
| ICD-10 | I69011 | Memory deficit following nontraumatic subarachnoid hemorrhage |
| ICD-10 | I69012 | Visuospatial deficit and spatial neglect following nontraumatic subarachnoid hemorrhage |
| ICD-10 | I69013 | Psychomotor deficit following nontraumatic subarachnoid hemorrhage |
| ICD-10 | I69014 | Frontal lobe and executive function deficit following nontraumatic subarachnoid hemorrhage |
| ICD-10 | I69015 | Cognitive social or emotional deficit following nontraumatic subarachnoid hemorrhage |
| ICD-10 | I69018 | Other symptoms and signs involving cognitive functions following nontraumatic subarachnoid hemorrhage |
| ICD-10 | I69019 | Unspecified symptoms and signs involving cognitive functions following nontraumatic subarachnoid hemorrhage |
| ICD-10 | I69020 | Aphasia following nontraumatic subarachnoid hemorrhage |
| ICD-10 | I69021 | Dysphasia following nontraumatic subarachnoid hemorrhage |
| ICD-10 | I69022 | Dysarthria following nontraumatic subarachnoid hemorrhage |
| ICD-10 | I69023 | Fluency disorder following nontraumatic subarachnoid hemorrhage |
| ICD-10 | I69028 | Other speech and language deficits following nontraumatic subarachnoid hemorrhage |
| ICD-10 | I69031 | Monoplegia of upper limb following nontraumatic subarachnoid hemorrhage affecting right dominant side |
| ICD-10 | I69032 | Monoplegia of upper limb following nontraumatic subarachnoid hemorrhage affecting left dominant side |
| ICD-10 | I69033 | Monoplegia of upper limb following nontraumatic subarachnoid hemorrhage affecting right non-dominant side |
| ICD-10 | I69034 | Monoplegia of upper limb following nontraumatic subarachnoid hemorrhage affecting left non-dominant side |
| ICD-10 | I69039 | Monoplegia of upper limb following nontraumatic subarachnoid hemorrhage affecting unspecified side |
| ICD-10 | I69041 | Monoplegia of lower limb following nontraumatic subarachnoid hemorrhage affecting right dominant side |
| ICD-10 | I69042 | Monoplegia of lower limb following nontraumatic subarachnoid hemorrhage affecting left dominant side |
| ICD-10 | I69043 | Monoplegia of lower limb following nontraumatic subarachnoid hemorrhage affecting right non-dominant side |
| ICD-10 | I69044 | Monoplegia of lower limb following nontraumatic subarachnoid hemorrhage affecting left non-dominant side |
| ICD-10 | I69049 | Monoplegia of lower limb following nontraumatic subarachnoid hemorrhage affecting unspecified side |
| ICD-10 | I69051 | Hemiplegia and hemiparesis following nontraumatic subarachnoid hemorrhage affecting right dominant side |
| ICD-10 | I69052 | Hemiplegia and hemiparesis following nontraumatic subarachnoid hemorrhage affecting left dominant side |
| ICD-10 | I69053 | Hemiplegia and hemiparesis following nontraumatic subarachnoid hemorrhage affecting right non-dominant side |
| ICD-10 | I69054 | Hemiplegia and hemiparesis following nontraumatic subarachnoid hemorrhage affecting left non-dominant side |
| ICD-10 | I69059 | Hemiplegia and hemiparesis following nontraumatic subarachnoid hemorrhage affecting unspecified side |
| ICD-10 | I69061 | Other paralytic syndrome following nontraumatic subarachnoid hemorrhage affecting right dominant side |
| ICD-10 | I69062 | Other paralytic syndrome following nontraumatic subarachnoid hemorrhage affecting left dominant side |
| ICD-10 | I69063 | Other paralytic syndrome following nontraumatic subarachnoid hemorrhage affecting right non-dominant side |
| ICD-10 | I69064 | Other paralytic syndrome following nontraumatic subarachnoid hemorrhage affecting left non-dominant side |
| ICD-10 | I69065 | Other paralytic syndrome following nontraumatic subarachnoid hemorrhage, bilateral |
| ICD-10 | I69069 | Other paralytic syndrome following nontraumatic subarachnoid hemorrhage affecting unspecified side |
| ICD-10 | I69090 | Apraxia following nontraumatic subarachnoid hemorrhage |
| ICD-10 | I69091 | Dysphagia following nontraumatic subarachnoid hemorrhage |
| ICD-10 | I69092 | Facial weakness following nontraumatic subarachnoid hemorrhage |
| ICD-10 | I69093 | Ataxia following nontraumatic subarachnoid hemorrhage |
| ICD-10 | I69098 | Other sequelae following nontraumatic subarachnoid hemorrhage |

**Supplemental Table 2.** ICD codes used to define head injury

| **ICD-9 Codes** | |
| --- | --- |
| 800.xx | Fracture of vault of skull |
| 801.xx | Fracture of base of skull |
| 803.xx | Other and unqualified skull fractures |
| 804.xx | Multiple fractures involving skull or face with other bones |
| 850.xx | Concussion |
| 851.xx | Cerebral laceration and contusion |
| 852.xx | Subarachnoid, subdural, and extradural hemorrhage following injury |
| 853.xx | Other and unspecified intracranial hemorrhage following injury |
| 854.xx | Intracranial injury of other and unspecified nature |
| 959.01 | Head injury, unspecified |
| **ICD-10 Codes** | |
| S02.0 | Fracture of vault of skull |
| S02.1X | Fracture of base of skull |
| S02.8 | Fractures of other unspecified skull and facial bones |
| S02.91 | Unspecified fracture of skull |
| S04.02 | Injury of optic chiasm |
| S04.03X | Injury of optic tract and pathways |
| S04.04X | Injury of visual cortex |
| S06.X | Intracranial injuries, concussion, traumatic cerebral edema, diffuse and focal traumatic brain injury, traumatic epidural, subdural, and subarachnoid hemorrhage |
| S07.1 | Crushing injury of skull |

**Table S3.** Annual incidence of non-traumatic subarachnoid hemorrhage per 100,000 discharges (95% confidence interval) in the National Inpatient Sample (NIS), 2012-2015.

|  | 2012 | 2013 | 2014 | 2015 | Change 2012-2015 |
| --- | --- | --- | --- | --- | --- |
| Overall | 68.7 (67.8, 69.5) | 70.4 (69.5, 71.3) | 72.9 (72.0, 73.8) | 79.8 (78.9, 80.7) | 3.6 (-0.1, 7.3) |
| Age |  |  |  |  |  |
| ≤24 years | 10.0 (9.3, 10.7) | 9.5 (8.8, 10.2) | 11.2 (10.5, 12.0) | 10.0 (9.3, 10.7) | 0.2 (-1.5, 1.8) |
| 25-49 years | 73.2 (71.4, 75.0) | 72.3 (70.5, 74.1) | 73.0 (71.1, 74.8) | 71.4 (69.6, 73.2) | -0.5 (-1.8, 0.8) |
| 50-74 years | 109.6 (107.7, 111.4) | 111.3 (109.4, 113.1) | 114.2 (112.3, 116.1) | 124.0 (122.1, 126.0) | 4.6 (-1.2, 10.4) |
| ≥75 years | 58.2 (56.5, 59.9) | 63.4 (61.6, 65.2) | 67.2 (65.3, 69.1) | 85.2 (83.1, 87.3) | 8.5 (-1.4, 18.4) |
| Missing | -- | 189.3 (93.6, 285.0) | -- | 107.2 (53.0, 161.4) | -- |
| Sex |  |  |  |  |  |
| Male | 61.0 (59.7, 62.2) | 64.8 (63.5, 66.1) | 67.9 (66.6, 69.2) | 76.7 (75.3, 78.0) | 5.0 (1.1, 8.9) |
| Female | 74.4 (73.2, 75.6) | 74.5 (73.3, 75.7) | 76.6 (75.4, 77.8) | 82.1 (80.9, 83.4) | 2.5 (-1.2, 6.2) |
| Missing | -- | 69.1 (8.5, 129.6) | -- | 91.4 (34.8, 148.0) | -- |
| Race or Ethnicity |  |  |  |  |  |
| White | 64.1 (63.1, 65.2) | 67.7 (66.6, 68.8) | 69.2 (68.1, 70.3) | 74.8 (73.6, 75.9) | 3.4 (1.0, 5.7) |
| Black | 69.0 (66.7, 71.3) | 69.5 (67.1, 71.8) | 75.2 (72.8, 77.7) | 83.0 (80.5, 85.4) | 4.8 (0.3, 9.8) |
| Hispanic | 71.4 (68.8, 74.0) | 66.9 (64.4, 69.4) | 70.3 (67.7, 72.9) | 82.1 (79.3, 84.8) | 3.6 (-7.5, 14.6) |
| Asian or Pacific Islander | 107.2 (100.5, 113.8) | 113.3 (106.5, 120.1) | 119.4 (112.5, 126.3) | 122.6 (115.8, 129.4) | 5.2 (3.1, 7.4) |
| Native American | 67.7 (57.5, 77.9) | 57.8 (47.4, 68.1) | 55.0 (44.9, 65.0) | 79.3 (67.4, 91.3) | 3.2 (-20.9, 27.3) |
| Other | 97.0 (91.7, 102.4) | 90.1 (84.6, 95.6) | 86.8 (81.5, 92.1) | 106.2 (100.1, 112.2) | 2.4 (-16.4, 21.2) |
| Missing | 77.7 (73.9, 81.4) | 78.0 (74.4, 81.7) | 83.4 (79.5, 87.3) | 85.5 (81.6, 89.4) | 2.9 (0.1, 5.7) |
| Region |  |  |  |  |  |
| Northeast | 65.4 (63.5, 67.3) | 62.5 (60.7, 64.4) | 69.7 (67.7, 71.7) | 78.4 (76.3, 89.4) | 4.6 (-3.7, 12.9) |
| Midwest | 61.8 (60.1, 63.5) | 69.1 (67.3, 70.9) | 71.1 (69.2, 72.9) | 75.8 (73.9, 77.8) | 4.4 (1.4, 7.4) |
| South | 68.9 (67.5, 70.3) | 69.5 (68.1, 70.9) | 71.8 (70.4, 73.2) | 79.3 (77.8, 80.7) | 3.4 (-1.5, 8.2) |
| West | 79.4 (77.4, 81.5) | 81.1 (79.0, 83.2) | 80.2 (78.1, 82.2) | 86.5 (84.4, 88.7) | 2.0 (-2.3, 6.4) |

**Table S4.** Annual incidence of non-traumatic subarachnoid hemorrhage per 100,000 discharges (95% confidence interval) in the National Inpatient Sample (NIS), 2016-2020.

|  | 2016 | 2017 | 2018 | 2019 | 2020 | Change 2016-2020 |
| --- | --- | --- | --- | --- | --- | --- |
| Overall | 108.1 (107.1, 109.2) | 104.6 (103.6, 105.7) | 101.3 (100.3, 102.4) | 99.1 (98.1, 100.2) | 96.6 (95.5, 97.6) | -2.9 (-3.4, -2.3) |
| Age |  |  |  |  |  |  |
| ≤24 years | 10.5 (9.7, 11.2) | 10.9 (10.1, 11.6) | 9.5 (8.8, 10.2) | 9.9 (9.2, 10.7) | 7.8 (7.1, 8.5) | -0.6 (-1.4, 0.1) |
| 25-49 years | 87.6 (85.6. 89.6) | 86.5 (84.5, 88.5) | 87.2 (85.1, 89.2) | 84.9 (82.9, 86.9) | 83.4 (81.4, 85.5) | -1.0 (-1.9, -0.1) |
| 50-74 years | 157.8 (155.6, 159.9) | 155.3 (153.2, 157.5) | 149.8 (147.6, 151.9) | 147.2 (145.1, 149.3) | 145.3 (143.2, 147.5) | -3.3 (-4.4, -2.2) |
| ≥75 years | 144.0 (141.3, 146.8) | 127.3 (124.8, 129.9) | 119.4 (116.9, 121.9) | 113.7 (111.3, 116.1) | 108.7 (106.2, 111.2) | -8.4 (-12.7, -4.2) |
| Missing | 70.3 (8.7, 131.9) | -- | -- | -- | -- | -- |
| Sex |  |  |  |  |  |  |
| Male | 105.4 (103.8, 107.0) | 100.3 (98.7, 101.9) | 98.5 (96.9, 100.0) | 93.6 (92.0, 95.1) | 92.4 (90.8, 94.0) | -3.3 (-4.4, -2.1) |
| Female | 110.3 (108.9, 111.8) | 108.0 (106.5, 109.4) | 103.6 (102.2, 105.0) | 103.6 (102.1, 105.0) | 100.0 (98.5, 101.4) | -2.5 (-3.6, -1.4) |
| Missing | 23.9 (2.9, 44.8) | 105.3 (13.0, 197.5) | 101.8 (12.6, 191.0) | -- | -- | -- |
| Race or Ethnicity |  |  |  |  |  |  |
| White | 102.3 (101.0, 103.6) | 97.3 (96.0, 98.6) | 94.8 (93.5, 96.0) | 92.9 (91.7, 94.2) | 93.5 (92.2, 94.9) | -2.2 (-4.1, -0.4) |
| Black | 110.0 (107.1, 112.8) | 108.3 (105.5, 111.1) | 106.1 (103.3, 108.9) | 106.9 (104.1, 109.7) | 101.4 (98.6, 104.2) | -1.9 (-3.4, -0.3) |
| Hispanic | 108.4 (105.2, 111.6) | 114.7 (111.5, 117.9) | 108.9 (105.8, 111.9) | 98.0 (95.0, 101.0) | 93.9 (91.0, 96.9) | -4.6 (-9.9, 0.8) |
| Asian or Pacific Islander | 187.1 (178.7, 195.4) | 168.0 (160.2, 175.8) | 145.2 (138.0, 152.4) | 148.4 (141.1, 155.7) | 140.3 (132.9, 147.7) | -11.3 (-20.2, -2.4) |
| Native American | 77.4 (65.7, 89.0) | 110.6 (96.8, 124.4) | 97.8 (85.0, 110.5) | 88.9 (77.0, 100.7) | 78.0 (66.7, 89.4) | -2.1 (-17.9, 13.8) |
| Other | 134.5 (127.8, 141.2) | 122.8 (166.6, 129.1) | 121.3 (115.2, 127.5) | 119.4 (113.2, 125.6) | 101.1 (95.2, 106.9) | -7.0 (-12.4, -1.7) |
| Missing | 115.5 (110.6, 120.4) | 111.3 (106.0, 116.6) | 115.9 (109.7, 122.0) | 123.2 (116.8, 129.7) | 99.7 (93.9, 105.6) | -2.0 (-11.3, 7.4) |
| Region |  |  |  |  |  |  |
| Northeast | 101.7 (99.2, 104.1) | 96.2 (93.8, 98.5) | 94.0 (91.7, 96.4) | 93.6 (91.2, 95.9) | 90.3 (87.9, 92.8) | -2.5 (-4.1, -1.0) |
| Midwest | 96.3 (94.1, 98.5) | 89.0 (86.9, 91.1) | 88.6 (86.6, 90.7) | 87.3 (85.2, 89.4) | 90.3 (88.1, 92.5) | -1.4 (-4.6, 1.9) |
| South | 109.4 (107.6, 111.1) | 108.1 (106.4, 109.8) | 103.3 (101.6, 105.0) | 100.6 (99.0, 102.3) | 97.2 (95.5, 98.9) | -3.2 (-4.0, -2.4) |
| West | 124.9 (122.4, 127.5) | 123.0 (120.4, 125.5) | 118.2 (115.7, 120.7) | 114.2 (111.8, 116.7) | 107.7 (105.1, 110.2) | -4.3 (-5.7, -2.9) |

**Table S5.** Joinpoint regression analysis of trends in SAH hospitalizations, 2012–2020^1,2^

|  | **Epoch 1** | | | **Epoch 2** | | |
| --- | --- | --- | --- | --- | --- | --- |
|  | **Years** | **Estimate**  (95% CI) | **p-value** | **Years** | **Estimate**  (95% CI) | **p-value** |
| **Overall** | 2012-2017 | 10.3 (2.4, 18.7) | 0.02 | 2017-2020 | -3.2 (-16.3, 11.9) | 0.57 |
| **Age** |  |  |  |  |  |  |
| ≤24 years | 2012-2017 | 1.7 (-5.2, 9.1) | 0.54 | 2017-2020 | -8.3 (-23.9, 10.4) | 0.26 |
| 25-49 years | 2012-2020 | 2.6 (0.7, 4.6) | 0.01 | -- | -- | -- |
| 50-74 years | 2012-2017 | 8.5 (2.0, 15.5) | 0.02 | 2017-2020 | -2.8 (-15.0, 11.2) | 0.59 |
| ≥75 years | 2012-2016 | 27.2 (20.5, 34.4) | <0.001 | 2016-2020 | -4.3 (-8.3, -0.1) | 0.05 |
| **Sex** |  |  |  |  |  |  |
| Male | 2012-2016 | 15.2 (12.4, 18.0) | <0.001 | 2016-2020 | -1.5 (-3.6, 0.7) | 0.13 |
| Female | 2012-2017 | 9.5 (1.0, 18.8) | 0.04 | 2017-2020 | -2.8 (-18.1, 15.3) | 0.67 |
| **Race/Ethnicity** |  |  |  |  |  |  |
| White | 2012-2016 | 12.0 (10.1, 14.1) | <0.001 | 2016-2020 | -0.4 (-2.0, 1.3) | 0.58 |
| Black | 2012-2017 | 11.5 (3.8, 19.7) | 0.01 | 2017-2020 | -2.6 (-15.2, 11.8) | 0.62 |
| Hispanic | 2012-2017 | 12.6 (2.8, 23.4) | 0.02 | 2017-2020 | -6.3 (-22.2, 12.9) | 0.39 |
| Asian or Pacific Islander | 2012-2016 | 14.8 (8.8, 21.1) | 0.002 | 2016-2020 | -4.8 (-9.0, -0.4) | 0.04 |
| Native American | 2012-2017 | 12.1 (0.5, 25.1) | 0.04 | 2017-2020 | -6.8 (-25.3, 16.3) | 0.43 |
| Other | 2012-2017 | 8.0 (-2.4, 19.4) | 0.10 | 2017-2020 | -6.3 (-25.5, 17.8) | 0.47 |
| **Region** |  |  |  |  |  |  |
| Northeast | 2012-2016 | 13.0 (7.1, 19.1) | 0.003 | 2016-2020 | -1.1 (-5.6, 3.6) | 0.55 |
| Midwest | 2012-2016 | 10.4 (7.4, 13.6) | 0.001 | 2016-2020 | -0.5 (-2.9, 2.0) | 0.63 |
| South | 2012-2017 | 11.5 (2.1, 21.7) | 0.03 | 2017-2020 | -3.8 (-20.6, 16.5) | 0.60 |
| West | 2012-2017 | 11.4 (0.5, 23.4) | 0.04 | 2017-2020 | -4.5 (-23.0, 18.6) | 0.59 |

1. We used joinpoint regression, a modeling approach that allows for estimation of time-specific trends from aggregated data including complex survey data, to characterize trends in non-traumatic SAH hospitalizations before and after the ICD transition.
2. Joinpoints for regression analysis are selected using a data-driven approach based on weighted Bayesian information criterion.

**Table S6.** Sensitivity analysis restricting follow-up to 2012–2019

|  | **2012-2020** | **2012-2015** | **2016-2020** |
| --- | --- | --- | --- |
| Primary Analysis | 88.9 (88.6, 89.3) | 72.9 (72.5, 73.4) | 102.1 (101.6, 102.5) |
| Sensitivity Analysis | 88.1 (87.7, 88.4) | 72.9 (72.5, 73.4) | 103.3 (102.8, 103.8) |

**Table S7.** Sensitivity analysis excluding ICD-10 codes I62.9 and I69

|  | **2012-2020** | **2012-2015** | **2016-2020** |
| --- | --- | --- | --- |
| Primary Analysis | 88.9 (88.6, 89.3) | 72.9 (72.5, 73.4) | 102.1 (101.6, 102.5) |
| Sensitivity Analysis | 88.9 (88.2, 89.7) | 72.9 (71.9, 73.9) | 102.1 (101.0, 103.1) |

**Table S8.** Sensitivity analysis excluding persons with a diagnosis of traumatic brain injury (TBI)

|  | **2012-2020** | **2012-2015** | **2016-2020** |
| --- | --- | --- | --- |
| Primary Analysis | 88.9 (88.6, 89.3) | 72.9 (72.5, 73.4) | 102.1 (101.6, 102.5) |
| Sensitivity Analysis | 89.0 (88.2, 89.7) | 72.9 (72.0, 73.9) | 102.1 (101.0, 103.1) |
